# Dynamic Functional Connectivity Change-Point Detection With Random Matrix Theory Inference

**DOI:** 10.3389/fnins.2021.565029

**Published:** 2021-05-04

**Authors:** Jaehee Kim, Woorim Jeong, Chun Kee Chung

**Affiliations:** ^1^Department of Statistics, Duksung Women's University, Seoul, South Korea; ^2^College of Sungsim General Education, Youngsan University, Gyeongnam, South Korea; ^3^Department of Neurosurgery, Seoul National University Hospital, Seoul, South Korea; ^4^Department of Brain and Cognitive Sciences, Seoul National University, Seoul, South Korea

**Keywords:** Tracy-Widom distribution, random matrix theory, fMRI, epilepsy, eigenvalue, dynamic functional connectivity, covariance, change-point

## Abstract

To study the dynamic nature of brain activity, functional magnetic resonance imaging (fMRI) data is useful including some temporal dependencies between the corresponding neural activity estimates. Recent studies have shown that the functional connectivity (FC) varies according to time and location which should be incorporated into the model. Modeling this dynamic FC (DFC) requires time-varying measures of spatial region of interest (ROI) sets. To know about the DFC, change-point detection in FC is of particular interest. In this paper, we propose a method of detecting a change-point based on the maximum of eigenvalues via random matrix theory (RMT). From covariance matrices for FC of all ROI's, the temporal change-point of FC is decided by an RMT approach. Simulation results show that our proposed method can detect meaningful FC change-points. We also illustrate the effectiveness of our FC detection approach by applying our method to epilepsy data where change-points detected are explained by the changes in memory capacity. Our study shows the possibility of RMT based approach in DFC change-point problem and in studying the complex dynamic pattern of functional brain interactions.

## 1. Introduction

Our brain is a complex network which consists of spatially distributed, but functionally linked regions that continuously share information with each other. Recent advanced functional neuroimaging studies have provided new tools to measure and to examine functional interactions between brain regions. These make the examination of DFC in the human brain and lead a development of theory associated with the complex FC structures. The fMRI data contains information about brain activity with complex spatial-temporal correlations. FC in the brain may be represented by these spatial dependence patterns and may reveal important characteristics of brain function and individual variations in cognition and behavior. FC is fundamentally a statistical concept, and is generally defined and assessed using statistical measures such as correlation (Biswall et al., 1995), and cross-coherence (Sun et al., 2004) in the temporal evolution of neural activity between distinct brain locations. Hutchison et al. (2013a,b) researched promise, issues, and interpretations as related with DFC.

Resting-state fMRI measures spontaneous fluctuations in the blood-oxygenation-level-dependent (BOLD) signal in gray matter regions. The fMRI connectivity approaches estimate statistical dependencies between gray matter activity arising from different regions (Biswall et al., 1995; Sato et al., 2006; Friston, 2011). For example, FC alterations are linked to depression, schizophrenia, and other illnesses (Mayberg et al., 1999; Jafria et al., 2008). Koike et al. (2011) provided that the default mode network (DMN) and subsystems might serve to integrate brain regions with performing function specific to each level of arousal.

Functional connectivity modelings are addressed with independent component analysis (ICA) (McKeown et al., 1998; Calhoun et al., 2001), pairwise correlation analysis (Supekar et al., 2008), and partial correlation analysis (Salvador et al., 2005). A key assumption of the aforementioned approaches is that functional connectivity remains stationary throughout the scanning session. However, many recent researches provide evidence of the dynamic nature of the brain's functional organization (Hellyer et al., 2014). Functional networks appear to fluctuate on a time scale and to reorganize under tasks during the scanning session (Chang and Glover, 2010).

Time-varying and dynamic nature of connectivity is considered in many recent neuroimaging studies. A well-known method for exploring time-varying connectivity is applying a sliding window of pre-specified length. This method calculates the correlations between distinct locations throughout the duration of the window at each step. Limitations with this sliding window approach, that the window length strongly influences the temporal smoothness or variability observed in the resulting correlations are discussed (Lindquist et al., 2014).

Identifying change-points is an important subject in neuroimaging study as it shows properties of brain networks as they relate to experimental stimuli and disease processes. Many change-point methods for fMRI signals have been proposed (Lindquist et al., 2007; Robinsona et al., 2010; Cribben et al., 2013; Xu and Lindquist, 2015). Xu and Lindquist (2015) assume that the timing of a subject's activation onset and duration are random variables, and their distributions are used to approximate the probability that a voxel/region is activated as a function of time with multi-subject change-point modeling. Bayesian approaches (Cheon and Kim, 2010; Kim and Cheon, 2010) and a non-parametric Fourier functional approach (Kim and Hart, 2011) are proposed to estimate change-points with distributional parameter changes which can be extended to the covariance change-point problems. Shao and Zhang (2010) study tests for change-point in time series, which is applicable to each individual fMRI series. Recently change-point detection for the multivariate covariance matrix are proposed for brain networks (Kim, 2019; Chenouri et al., 2020).

There have been many studies on testing for distributional structure changes for a sequence of independent and identically distributed (IID) random variables, including Csörgő and Horváth (1997). However test statistics developed for change-point detection in the IID context may not work with the time series observed in neuroimaging as they do not take into account the temporal dependence in the data. Aue et al. (2009) proposed break detection in covariance based on the *vech* operator to summarize information in the covariance matrix. Here *vech* is the vectorization operator which converts the matrix into a column vector. Aue and Horváth (2013) reviewed break points estimation in covariance structure in time series. Inclán and Tiao (1994) proposed variance change detection with cumulative sums for independent series. Petersen and Muller (2016) studied functional data covariance and applied to Alzheimer fMRI data. For detecting correlation change, Wied and Krömer (2012) considered the cumulated sums of empirical correlations to make a change test.

In the present study, we aim to deal with spatial time series data to detect FC change-points based on random matrix theory. Our approach does not make any parametric assumptions. It is especially designed for detecting one change-point but is extendable to multiple change-points in FC. In addition, we applied our developed method to fMRI data of post-surgical epilepsy patients. Previous studies have shown that activity and FC of the medial temporal lobe (MTL) during the post-encoding offline period are closely related to the memory consolidation (Tambini et al., 2010; Staresina et al., 2013; Tambini and Davachi, 2013). However, how the brain supports the memory consolidation process in the absence of one MTL structure has not been investigated. Here, we applied our developed method to the post-encoding rest period to evaluate its usefulness in characterization of memory consolidation networks in MTL resected brains.

This paper is organized as follows. In section 2, we begin by looking at the covariance change-point problem with the proposed statistics. Simulation studies are shown in section 3. Application of the proposed method to the experimental epilepsy data is shown in section 4. The paper concludes with a discussion.

## 2. Methods

### 2.1. Statistical Problems for Dynamic Functional Connectivity

A natural unit of fMRI analysis is multi-voxel regions of the brain that change their level of activation. The fMRI data can be widely used to detect and delineate regions of the brain that change in response to specific stimuli and tasks. We represent a single scan from a subject as a 3-D rectangular lattice consisting of volume elements (voxels). From one scanning session, each voxel contains serial measures of localized brain activity called blood-oxygen-level dependent (BOLD) fMRI responses. Through the hemodynamic response (HR) process, blood releases oxygen to neurons at a greater rate than to inactive neurons. This causes a change of the relative levels of oxyhemoglobin and deoxyhemoglobin that is detectable due to magnetic susceptibility. Since the number of intracranial voxels is too large to estimate a global spatial correlation matrix including all voxel pairs, we partition the voxels into mutually exclusive neuroanatomical regions and select ROIs in order to make the ROI-level inference.

Let *y*_*ivt*_ be an observation measured at voxel or region ***v*** from the serial fMRI BOLD responses for an ***i***th subject. Let *i* = 1, …, *N* represent subjects, *v* = 1, …, ***d*** voxels (or ROIs), and *t* = 1, …, *T* time points. For the *i*th subject, *d* × *T* observed data matrix is obtained. It makes a multivariate time series for each subject and its covariance change is of interest. Tests that assess the structural stability of volatilities or cross-volatilities for multivariate non-linear time series such as fMRI data and change-point estimation is of great importance for understanding the underlying dynamics.

Let's fix the *i*th subject for the notational simplicity. Consider the observed **y**_*t*_ is a ***d*** × 1 random vector at the time point *t* with $E\left[\|y_{t}{\|}^{2}\right]<\infty$. Here ‖ · ‖ denotes the Euclidean norm in ℝ^*d*^. Based on the observations of the random vector **y**_1_, …, **y**_*T*_, consider a test to identify covariance matrix change for each subject.

The null hypothesis is

(1) $$\begin{array}{l} {\text{H}}_{0}:Cov\left(y_{1}\right)=\cdots =Cov\left(y_{T}\right) \end{array}$$

which indicates the constancy of the covariances during the observational time period. Against this null hypothesis the alternative hypothesis with at most one change in the covariance is

(2) $$\begin{array}{l} {\text{H}}_{A}:Cov\left(y_{1}\right)=\cdots =Cov\left(y_{\tau }\right)\neq Cov\left(y_{\tau +1}\right)=\cdots =Cov\left(y_{T}\right) \end{array}$$

where *τ* is the change-point. Let **Σ**_*t*_ = *Cov*(**y**_*t*_).

### 2.2. Dynamic Functional Connectivity Test With the Largest Eigenvalues

We summarize the covariance structure using maximum eigenvalues, defined as the largest eigenvalues of the covariance matrix as a functional representative measure of the matrix.

Let λ_1_(*t*) = max{λ_1_(*t*), …, λ_*d*_(*t*)} denote the largest eigenvalues of the matrix **Σ**_*t*_. The null hypothesis in (1) can be considered as that

(3) $$\begin{array}{l} {\text{H}}_{0}:\lambda_{1}\left(1\right)=\cdots =\lambda_{1}\left(T\right) \end{array}$$

which indicates the constancy of the maximum eigenvalues of covariances during the observation time period. The alternative hypothesis can be written with the maximum eigenvalue measure as

(4) $$\begin{array}{l} {\text{H}}_{A}:\lambda_{1}\left(1\right)=\cdots =\lambda_{1}\left(\tau \right)\neq \lambda_{1}\left(\tau +1\right)=\cdots =\lambda_{1}\left(T\right) \end{array}$$

where *τ* is denoted as the change-point.

Suppose that the data are from *d*−dimensional Gaussian distribution with mean zero and positive definite covariance matrix **Σ**. The sample covariance matrix (SCM) formed with these *T* samples as

(5) $$\begin{array}{l} {\text{S}}_{T}=\frac{1}{T}\overset{T}{\underset{u=1}{\sum }}y_{u}y_{u}^{\prime } \end{array}$$

has the (central) Wishart distribution (Goodman, 1963; Raj Rao et al., 2008).

Estimating the eigenvalues of a population covariance matrix from a sample covariance matrix is a problem of fundamental importance in multivariate statistics since the eigenvalues of covariance matrices play a key role in many widely used techniques with a suitable theory (Goodman, 1963; Okamoto, 1973; Gupta and Nagar, 1999; Mohsen, 2013). The amount of variance explained is measured by the eigenvalues of the population covariance, **Σ**_*d* × *d*_. Random matrix theory predicts that the eigenvalues of a sample covariance matrix **S**_*T*_ are not good estimators of the eigenvalues of the population covariance when the sample size *T* and the number of variables *d* are large. El Karoui (2008) proposed a better estimator for the eigenvalues of large dimensional covariance matrices. Under the weak assumption that the Marčenko and Pastur (1967) equation holds, the El Karoui (2008) estimator can be thought as a shrinkage in a non-linear fashion.

Let λ_1_ ≥ λ_2_ ≥ ⋯ ≥ λ_*d*_ be the eigenvalues of the population covariance matrix. Accordingly let *l*_1_ ≥ *l*_2_ ≥ ⋯ ≥*l*_*d*_ be the eigenvalues of the sample covariance matrix. Anderson (1963) showed that the case where **Σ** = **I**_*d*×*d*_ with all the population eigenvalues one, under some moment conditions, the maximum eigenvalue is not a consistent estimator of λ_1_ and can be extremely biased when *T* and *d* are both large. To de-bias extreme sample eigenvalues, we use population spectral distribution, a probability measure that characterizes the population eigenvalues (El Karoui, 2007). The limiting distribution of the sample eigenvalues are dependent on data dependency and their covariance matrix. Johnstone (2001) derived the limiting distribution of the largest sample eigenvalue with independent Gaussian case and Bai and Silverstein (2010) dealt with this problem the sub-Gaussian case. In terms of the spectral decomposition of a covariance matrix, an estimator is rotation-equivariant if and only if the eigenvectors are the same as those of the sample covariance matrix (Mohsen, 2013). Thus, the differences between two such estimators appear only in their eigenvalues.

### 2.3. Dynamic Functional Connectivity Test With Tracy-Widom Distributions

Assume that **y**_*u*_'s are independent from ***d***−dimensional distribution, *u* = 1,…, *T*. Let the sample covariance matrix up to *t* and after *t* be

(6) $$\begin{array}{ll} {\text{A}}_{t} & =\overset{t}{\underset{u=1}{\sum }}\left(y_{u}-{\overset{-}{y}}_{t}\right){\left(y_{u}-{\overset{-}{y}}_{t}\right)}^{\prime } \end{array}$$

(7) $$\begin{array}{ll} {\text{B}}_{t} & =\overset{T}{\underset{u=t+1}{\sum }}\left(y_{u}-{\overset{-}{y}}_{t*}\right){\left(y_{u}-{\overset{-}{y}}_{t*}\right)}^{\prime } \end{array}$$

where

(8) $$\begin{array}{l} {\overset{-}{y}}_{t}=\frac{1}{t}\overset{t}{\underset{u=1}{\sum }}y_{u}\text{ and }{\overset{-}{y}}_{t*}=\frac{1}{\left(T-t\right)}\overset{T}{\underset{u=t+1}{\sum }}y_{u} \end{array}$$

are the sample mean vectors separated by *t*. If **y**_*u*_'s are independent from *d*−dimensional normal distribution, **A**_*t*_ and **B**_*t*_ are *d* × *d* matrices following Wishart distributions denoted by **A**_*t*_ ~ *W*_*d*_(**Σ**, *t*) and **B**_*t*_ ~ *W*_*d*_(**Σ**, *T* − *t*) respectively under H_0_.

Consider that *t*, *T* − *t* ≥ *d*. The scale matrix **Σ** has no effect on the distribution of the eigenvalues, and so consider the largest eigenvalue λ_1_(*t*) of ${\left({\text{A}}_{t}+{\text{B}}_{t}\right)}^{-1}{\text{A}}_{t}$ as the greatest root statistic. If there exists change and change-point occurs at time point t, there is change in *A*_*t*_ and *B*_*t*_. This change is reflected in the test statistic *G*_*t*_.

Under *H*_0_ in (3), it holds that λ_1_(t*) = ⋯ = λ_1_(*T* − t*). For example, we can set t* = 30 to have enough degrees of freedom. We consider the following statistic to provide information about change and change-points in the covariance matrix.

(9) $$\begin{array}{l} G_{t}=\underset{t}{\max}eigen\left[{\left({\text{A}}_{t}+{\text{B}}_{t}\right)}^{-1}{\text{A}}_{t}\right]-\underset{t}{\max}eigen\left[{\left({\text{A}}_{t}+{\text{B}}_{t}\right)}^{-1}{\text{B}}_{t}\right]. \end{array}$$

Under *H*_0_, *E*[*G*_*t*_] = 0. Since **A**_*t*_ and **B**_*t*_ are positive definite, 0 < *ϕ* < 1. Equivalently *ϕ* is the largest root of the determinantal equation

(10) $$\begin{array}{l} det\left[{\text{A}}_{t}-\phi \left({\text{A}}_{t}+{\text{B}}_{t}\right)\right]=0. \end{array}$$

Johnstone (2009) provided the appropriate centering and scaling, and the logit transform *W*_*t*_ = *logit*(*ϕ*) = log(*ϕ*/(1 − *ϕ*)) is approximately Tracy-Widom distributed:

(11) $$\begin{array}{l} \frac{W_{t}-\mu_{t}}{\sigma_{t}}\Rightarrow Z_{1}\sim F_{1}. \end{array}$$

The distribution *F*_1_ was found by Tracy and Widom (1996) as the limiting law of the largest eigenvalue of *d* × *d* Gaussian symmetric matrix. The centering and scaling parameters are given by

(12) $$\begin{array}{l} \mu_{t}=2\log\tan\left(\frac{\varphi +\gamma }{2}\right),\\ \sigma_{t}^{3}=\frac{16}{{\left(t+(T-t)-1\right)}^{2}}\cdot \frac{1}{\sin^{2}(\varphi +\gamma )\sin\varphi \sin\gamma } \end{array}$$

where the angle parameters *φ* = *φ*_*t*_, *γ* = *γ*_*t*_ are defined by

(13) $$\begin{array}{l} \sin\left(\frac{\gamma }{2}\right)=\frac{\min(d,t)-1/2}{t+(T-t)-1},\\ \sin\left(\frac{\varphi }{2}\right)=\frac{\max(d,t)-1/2}{t+(T-t)-1}. \end{array}$$

We apply Tracy and Widom scaling and centering for each maximum eigenvalues of ${\left({\text{A}}_{t}+{\text{B}}_{t}\right)}^{-1}{\text{A}}_{t}$ and then $\max eigen\left[{\left({\text{A}}_{t}+{\text{B}}_{t}\right)}^{-1}{\text{B}}_{t}\right]$ respectively.

Therefore our test is modified by Tracy and Widom correction to de-bias of the eigenvalues as follows

(14) $$\begin{array}{l} G_{t}=\max eigen^{TW}\left[{\left({\text{A}}_{t}+{\text{B}}_{t}\right)}^{-1}{\text{A}}_{t}\right]-\max eigen^{TW}\left[{\left({\text{A}}_{t}+{\text{B}}_{t}\right)}^{-1}{\text{B}}_{t}\right]. \end{array}$$

Here max *eigen*^*TW*^ denotes that the maximum eigenvalue is modified by Tracy and Widom correction. We propose the test statistic for change as

(15) $$\begin{array}{l} \Lambda_{T}=\underset{1<t<T}{\max}G_{t}^{2} \end{array}$$

which rejects H_0_ if Λ_*T*_ is large. Accordingly the proposed change-point estimator is

(16) $$\begin{array}{l} \hat{\tau }=\text{arg}\underset{1<t<T}{\max}G_{t}^{2}. \end{array}$$

Permutation tests have the flexible class of tests for both non-parametric and parametric (model-based). The proposed statistics can be used regardless of the data distributions using this permutation approach. The threshold calculation based on block permutation can be applied for testing for change-points (Strasser and Weber, 1999; Husková, 2004). Exchangeability of the errors might be too strong of an assumption for time series applications due to the dependence structure of the observations. Block permutation is an applicable alternative. Block permutation principles are suitable for testing autoregressive series and they are refined (Kirch and Steinebach, 2006; Kirch, 2007; Zeileis and Hothorn, 2013). Approximations of critical values of the change test statistics can be obtained through block permutation methods (Husková, 2004; Luger, 2006; Kirch, 2007).

To do our testing procedure, the approximate critical values are required. For each subject with dependent time series data, each critical value can be obtained according to the block permutation tests. This block permutation tests provide asymptotically valid results in the presence of dependency.

## 3. Simulations

### 3.1. Simulation Study

In this section we set up the simulation in order to assess and compare the performance of the proposed estimator for change-point estimation. The simulation study is focused on change-point estimation with multi-subject data from the multivariate vector autoregressive (MVAR) models. The objective of each simulation is to detect the functional connectivity change-point.

Before change-point estimation, testing for change is done. Since it is not possible to obtain the exact distribution of the test statistic Λ_*T*_ analytically, it is determined by simulation. The data are synthetically generated from multivariate normal distribution *N*(**0**, **Σ**_*d*_) for the null distribution with the given dimension *d* as the number of ROIs, and the number of time points *T*. Several types of **Σ**_*d*_ are designed. For the comparison we also consider the Aue et al. (2009) method.

Aue et al. (2009) proposed the test for covariance change based on

(17) $$\begin{array}{l} S_{t,A}=\frac{1}{\sqrt{T}}\left(\sum_{j=1}^{t}\text{vech}[y_{j}y_{j}^{\;\prime }]-\frac{t}{T}\sum_{j=1}^{T}\text{vech}[y_{j}y_{j}^{\;\prime }]\right),\\ \;t=1,\ldots ,T. \end{array}$$

Their change test statistic is

(18) $$\Lambda_{\text{A}}=\max_{1<t<T}{S^{\prime }}_{t,A}{\hat{\Sigma }}_{T}^{-1}S_{t,A}\text{ with }{\hat{\Sigma }}_{T}=\hat{C}ov(\text{vech}[y_{j}y_{j}^{\;\prime }])$$

where ${\hat{\Sigma }}_{T}$ is a consistent covariance estimator. Aue et al. (2009) change-point estimator is

(19) $${\hat{\tau }}_{A}=\text{arg}\underset{1<t<T}{\max}|{S^{\prime }}_{t,A}{\hat{\Sigma }}_{T}^{-1}S_{t,A}|.$$

We use ${\hat{\Sigma }}_{T}$ as the sampled version of the moment estimator. The asymptotic critical values of the test statistics are computed from the block permutation based empirical distribution.

We simulate data according to MVAR(1) and MVAR(2) models. with the regional dimension *d* = 5 and *T* = 200 time points in 1, 000 repetitions. Since the brain network is high-dimensional, we also consider *d* = 20. The empirical critical values under the null distribution are obtained from no change-point model using block permutations for each subject. The fraction of one change-point is considered as *θ* = *τ*/*T* = 0.5 and 0.7 in the simulated data. We calculate the performance measure such as mean, median, square root of MSE (mean squared error), and the proportion around the true change-point as $p5=\hat{P}\left(|\hat{\tau }-\tau |<5\right)$ with 95 % confidence level.

Data are generated from the following model, **y**_*u*_ ~ MVAR(p) as

(20) $$\begin{array}{l} y_{u}=\overset{p}{\underset{k=1}{\sum }}{\text{B}}_{k}y_{u-k}+\epsilon_{u}, \end{array}$$

where **B**_*k*_ is *k*th parameter coefficient matrix of *MVAR*(*p*), *k* = 1, …, *p*. And **Σ**_1_ and **Σ**_2_ are covariance matrix of **ϵ**_*u*_'s before and after the change-point respectively. We consider *MVAR*(1) models in cases (i) and (ii), and *MVAR*(2) models in (iii) and (iv) in the followings.

- MVAR(1) with coefficient matrix **B**_1_
  (21) $$B_{1}=\{\begin{array}{ll} \Psi_{1}=[(0.2)1,0,\cdots ,0], & \;u\leq \tau \\ \Psi_{2}=\Psi_{1}+(0.1)I, & \;u>\tau . \end{array}$$
  where **I** is the identity matrix and **J** is a matrix whose components are all 1's.

- MVAR(1) with |**Σ**_1_| < |**Σ**_2_|
  (22) $$\{\begin{array}{ll} \Sigma_{1}=(0.5)I, & \;u\leq \tau \\ \Sigma_{2}=(1.0)I+(0.1)J-(0.1)I, & \;u>\tau . \end{array}$$
- MVAR(1) with |**Σ**_1_| > |**Σ**_2_|
  (23) $$\{\begin{array}{ll} \Sigma_{1}=(1.0)I+(0.1)J-(0.1)I, & \;u\leq \tau \\ \Sigma_{2}=(0.5)I, & \;u>\tau . \end{array}$$

- II. MVAR(2) with coefficient matrices **B**_1_ and **B**_2_
  (24) $$\begin{array}{l} B_{1}=\{\begin{array}{ll} \Psi_{11}=[(0.2)1,0,\cdots ,0], & u\leq \tau \\ \Psi_{12}=\Psi_{11}+(0.1)I, & u>\tau , \end{array}\\ B_{2}=\{\begin{array}{ll} \Psi_{21}=[(0.1)1,0,\cdots ,0], & \;u\leq \tau \\ \Psi_{22}=\Psi_{21}+(0.1)I, & \;u>\tau . \end{array} \end{array}$$

(iii) MVAR(2) with |**Σ**_1_| < |**Σ**_2_|
  (25) $$\{\begin{array}{ll} \Sigma_{1}=(0.5)I+(0.01)J-(0.01)I, & \;u\leq \tau \\ \Sigma_{2}=(1.0)I+(0.1)J-(0.1)I, & \;u>\tau , \end{array}$$
(iv) MVAR(2) with |**Σ**_1_| > |**Σ**_2_|
  (26) $$\{\begin{array}{ll} \Sigma_{1}=(1.0)I+(0.1)J-(0.1)I, & \;u\leq \tau \\ \Sigma_{2}=(0.5)I+(0.01)J-(0.01)I, & \;u>\tau . \end{array}$$

To illustrate the potential to detect multiple change-points, we can apply the procedure with the subsegment after the change-point. Each time we reject H_0_, we re-apply the method to the subsamples splitted by the proposed change-point.

We considered MVAR(1) and MVAR(2) cases with the different covariance structure after the change-point at *θ* = 0.5, 0.7. Tables 1, 2 provide MVAR(1) cases in which our estimators have smaller MSE's and higher matching proportions than Aue estimators. Tables 3, 4 give the change-point estimation results in MVAR(2) cases. Our method performs better than the Aue method in Tables 3, 4. Aue method depends on the covariance estimator ${\hat{\Sigma }}_{T}$ in the statistic in (19), and the change-point estimation results depend on the covariance structure accordingly. But our change-point estimator gives stable simulation results in the performance based upon mean and MSE. Overall in this simulation the proposed estimator performs very well.

**Table 1:** Change-point estimation results of the proposed $\hat{\theta }$ and Aue estimator ${\hat{\theta }}_{A}$ in case (i) MVAR(1) with *T* = 200 in 1, 000 repetitions.

	***d*** ***= 5***				***d*** ***= 20***			
**True**	***θ*** ***= 0.5***		***θ*** ***= 0.7***		***θ*** ***= 0.5***		***θ*** ***= 0.7***	
**Statistics**	$\hat{\theta }$	${\hat{\theta }}_{A}$	$\hat{\theta }$	${\hat{\theta }}_{A}$	$\hat{\theta }$	${\hat{\theta }}_{A}$	$\hat{\theta }$	${\hat{\theta }}_{A}$
Mean	0.495	0.666	0.692	0.730	0.499	0.683	0.690	0.753
Median	0.500	0.670	0.700	0.750	0.500	0.680	0.695	0.750
$\sqrt{MSE}$	0.052	0.194	0.062	0.109	0.048	0.194	0.052	0.065
p5	0.648	0.069	0.642	0.197	0.616	0.001	0.607	0.269

**Table 2:** Change-point estimation results of the proposed $\hat{\theta }$ and Aue estimator ${\hat{\theta }}_{A}$ in case (ii) MVAR(1) with *T* = 200 in 1, 000 repetitions.

	***d*** ***= 5***				***d*** ***= 20***			
**True**	***θ*** = ***0.5***		***θ*** = ***0.7***		***θ*** = ***0.5***		***θ*** = ***0.7***	
**Statistics**	$\hat{\theta }$	${\hat{\theta }}_{A}$	$\hat{\theta }$	${\hat{\theta }}_{A}$	$\hat{\theta }$	${\hat{\theta }}_{A}$	$\hat{\theta }$	${\hat{\theta }}_{A}$
Mean	0.509	0.419	0.689	0.734	0.503	0.327	0.691	0.756
Median	0.500	0.390	0.700	0.752	0.500	0.330	0.700	0.755
$\sqrt{MSE}$	0.062	0.207	0.063	0.104	0.052	0.182	0.052	0.068
p5	0.607	0.076	0.633	0.203	0.599	0.002	0.615	0.252

**Table 3:** Change-point estimation results of the proposed $\hat{\theta }$ and Aue estimator ${\hat{\theta }}_{A}$ in case (iii) MVAR(2) with *T* = 200 in 1, 000 repetitions.

	***d*** ***= 5***				***d*** ***= 20***			
**True**	***θ*** ***= 0.5***		***θ*** ***= 0.7***		***θ*** ***= 0.5***		***θ*** ***= 0.7***	
**Statistics**	$\hat{\theta }$	${\hat{\theta }}_{A}$	$\hat{\theta }$	${\hat{\theta }}_{A}$	$\hat{\theta }$	${\hat{\theta }}_{A}$	$\hat{\theta }$	${\hat{\theta }}_{A}$
Mean	0.500	0.624	0.689	0.735	0.498	0.621	0.691	0.736
Median	0.500	0.615	0.695	0.730	0.500	0.615	0.695	0.725
$\sqrt{MSE}$	0.052	0.148	0.054	0.053	0.051	0.146	0.049	0.054
p5	0.580	0.132	0.586	0.463	0.630	0.143	0.618	0.477

**Table 4:** Change-point estimation results of the proposed $\hat{\theta }$ and Aue estimator ${\hat{\theta }}_{A}$ (iv) MVAR(2) with *T* = 200 in 1, 000 repetitions.

	***d*** ***= 5***				***d*** ***= 20***			
**True**	***θ*** ***= 0.5***		***θ*** ***= 0.7***		***θ*** ***= 0.5***		***θ*** ***= 0.7***	
**Statistics**	$\hat{\theta }$	${\hat{\theta }}_{A}$	$\hat{\theta }$	${\hat{\theta }}_{A}$	$\hat{\theta }$	${\hat{\theta }}_{A}$	$\hat{\theta }$	${\hat{\theta }}_{A}$
Mean	0.502	0.554	0.699	0.622	0.505	0.574	0.694	0.629
Median	0.500	0.475	0.700	0.640	0.500	0.480	0.700	0.645
$\sqrt{MSE}$	0.056	0.193	0.048	0.110	0.059	0.209	0.057	0.104
p5	0.600	0.171	0.599	0.251	0.566	0.140	0.608	0.243

## 4. Application to the Epilepsy fMRI Data

### 4.1. Epilepsy fMRI Data

The same subjects from our previous study are used in this study except for one patient who showed a severe movement artifact (Jeong et al., 2019). Patients who underwent unilateral MTL resection (MTLR) to treat medically intractable temporal lobe epilepsy at Seoul National University Hospital at least 1 year before recruitment and were between 19 and 50 years of age are retrospectively recruited. We only include subjects who showed at least a low average or a higher level (scores> 80) of memory capacity and general intelligence evaluated by the standard neuropsychological test. A total of 34 patients (16 left and 18 right; median age = 32.5 years) and 24 age- and education-year-matched healthy controls (HC, median age = 32 years) are included in the present study. All subjects provided informed consent. This study was approved by the Institutional Review Board of Seoul National University Hospital.

The MR images were acquired on a research-dedicated 3T Magnetom Trio Tim Syngo (Siemens, Erlangen, Germany) using a 32-channel head coil. Five minutes of resting-state functional data (eyes open, fixation cross) were acquired both before (pre-encoding baseline) and after performing the in-scanner memory encoding paradigm of words and figures (post-encoding) using a T2^*^-weighted gradient echo planar imaging sequence (36 axial slices, slice thickness = 3.4 mm, no gap, TR = 2,000 ms, TE = 30 ms, FOV the change-point 220 × 220 mm, flip angel = 80°, voxel size = 3.4 × 3.4 × 3.4*mm*^3^, and interleaved). Whole-brain high-resolution anatomic T1-weighted images were obtained with the 3D TFL sequence (TR = 1,670 ms, TE = 1.89 ms, FOV = 250 × 250H mm, flip angle = 9°, voxel size = 1.0 × 1.0 × 1.0*mm*^3^). The resting-state functional data underwent a number of preprocessing steps including motion correction, slice time correction, co-registration, spatial normalization, and spatial smoothing (AFNI, version: 16.0.00, https://afni.nimh.nih.gov/afni/). More details about the memory encoding paradigm and data preprocessing have been described elsewhere (Jeong et al., 2018, 2019).

Since previous studies have shown that FC during the post-encoding resting-state is related to the memory performance (Tambini et al., 2010; Staresina et al., 2013; Tambini and Davachi, 2013), we thought that data that showed evident FC changes of memory related areas rather than entire data of post-encoding resting-state may possibly provide the additional information of the memory consolidation network. In order to find the point that showed evident FC changes, we used change-point analysis.

For change-point estimation analysis, we select 20 ROIs within DMN, which is a well-known network that subserves memory function (Andrews-Hanna et al., 2010; Jeong et al., 2015). Table 5 shows the details of selected ROIs. In both pre- and post-encoding rest data, BOLD signals were extracted within each selected ROI (8 mm radius). For patients, one ROI, left hippocampus for left MTLR (LMTLR) and right hippocampus for right MTLR (RMTLR), was excluded from BOLD time series extraction. The extracted time series are then used for change-point estimation of post-encoding rest data to detect changes of FC after memory encoding. Next, pair-wise Pearson correlation coefficients between each pair of ROIs are calculated and averaged for each ROI, separately for time series after change-point and pre-encoding. FC is additionally calculated for the whole-segment of post-encoding time series in order to evaluate the usefulness of the change-point estimation method in the detection of memory encoding-related FC changes.

**Table 5:** Regions of Interest.

**ROI**	**No**		**Region**	**Abbreviation**	**MNI**	**Coordinates**	
**Left**		**Right**			**x**	**y**	**z**
	1		Dorsal medial prefrontal cortex	dMPFC	0	52	26
2		11	Anterior medial prefrontal cortex	aMPFC	–6	52	–2
3		12	Hippocampal formation	HF	–22	–20	–26
4		13	Lateral temporal cortex	LTC	–60	–24	–18
5		14	Posterior cingulate cortex	PCC	–8	–56	26
6		15	Parahippoampal cortex	PHC	–28	–40	–12
7		16	Posterior inferior parietal lobule	pIPL	–44	–74	32
8		17	Retrosplenial cortex	Rsp	–14	–52	8
9		18	Temporal pole	TempP	–50	14	–40
10		19	Temporal parietal junction	TPJ	–54	–54	28
	20		Ventral medial prefrontal cortex	vMPFC	0	26	–18

### 4.2. Change Analysis for Epilepsy Data

Change analysis starts with an individual test for existence of change. Under the significance of change, change-point estimation can be accomplished for its identification. With the epilepsy data, we performed the block permutation method to obtain the critical values. For dependent data like fMRI data, block permutation is preferable and useful (Kirch and Steinebach, 2006; Kirch, 2007; Adolf et al., 2011). This block permutation procedure is done individually because the change-point should be estimated subject-wise. The block permutation critical values are obtained with the block size *K* = 5 in 1,000 repetitions. At the significance level *α* = 0.05 we reject the null hypothesis for each individual subject except hc22 and lt16 (significant at *α* = 0.10). Since we focus on detectable change-point estimation, we get and use the change-points for all subjects. The change-point is estimable and informative for change, so we include these two subjects for the analysis. Therefore, we identify one change-point for each subject.

**Figure 1:**
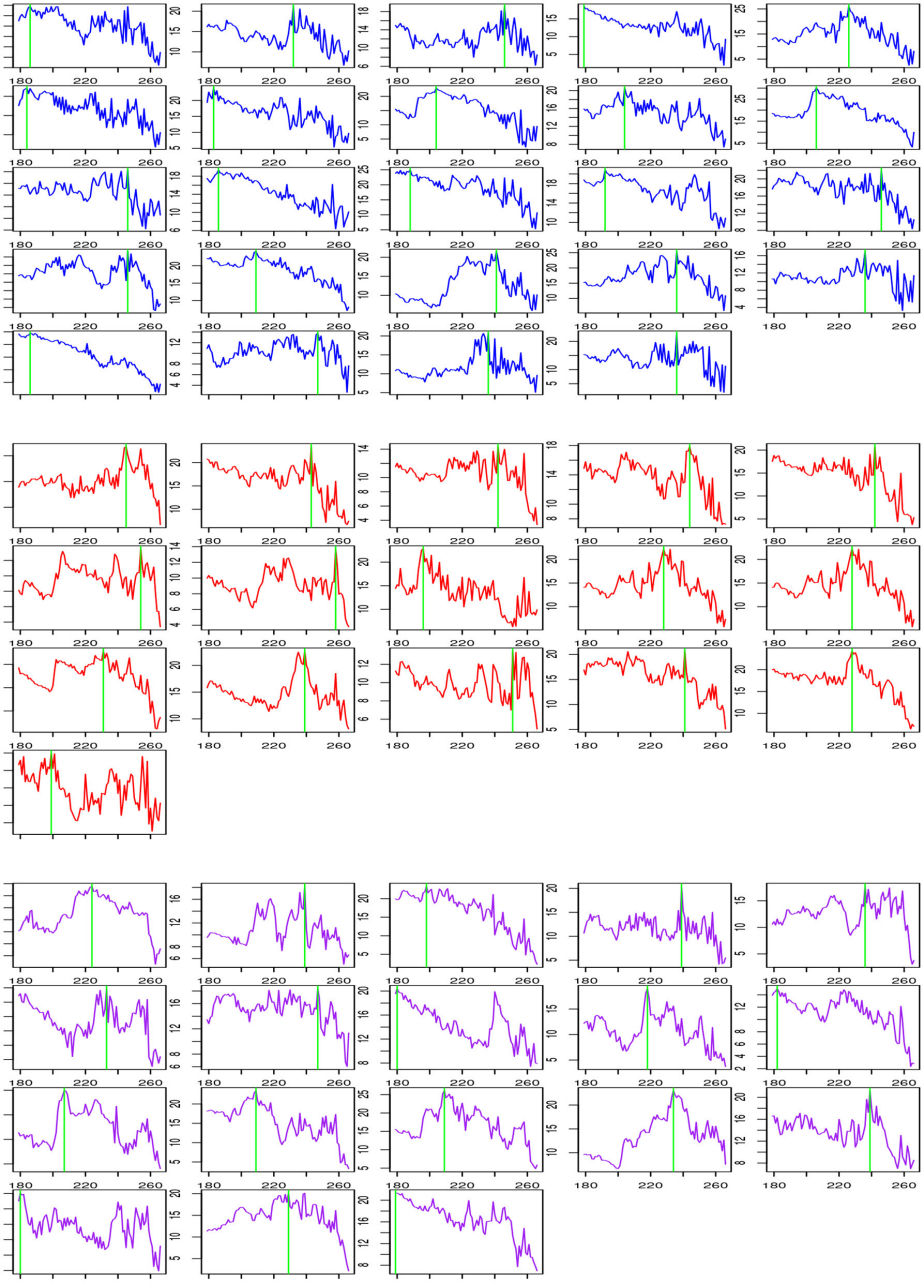
Change process $G_{t}^{2}$ according *t* and change-point estimation for each subject during post-encoding rest in HC **(Upper)**, LMTLR **(Middle)**, and RMTLR **(Lower)** groups.

Table 6 shows a summary of change-point estimation and Figure 1 shows the change-points of individual subjects. The experiments are conducted during the two resting states. Change-point estimation is done during the second state because the first resting state is considered the baseline. Change-points are significantly different among groups [*F*_(2,55)_ = 4.29, *p* < 0.05, ANOVA]. Bonferroni *post-hoc* tests reveal that the LMTLR group shows significantly higher change-point values than the HC (*p* < 0.05) and RMTLR groups (*p* < 0.05). There is no significant differences between the HC and RMTLR groups (*p* > 0.10).

**Table 6:** Change-point estimation results for post-encoding rest of epilepsy data set.

	**Change-point mean**	**SD**
HC	215.87	25.64
LMTLR	235.56	17.35
RMTLR	215.67	23.53

*HC, healthy controls; LMTLR, patients with left medial temporal lobe resection;*

*RMTLR, patients with right medial temporal lobe resection*.

**Figure 2:**
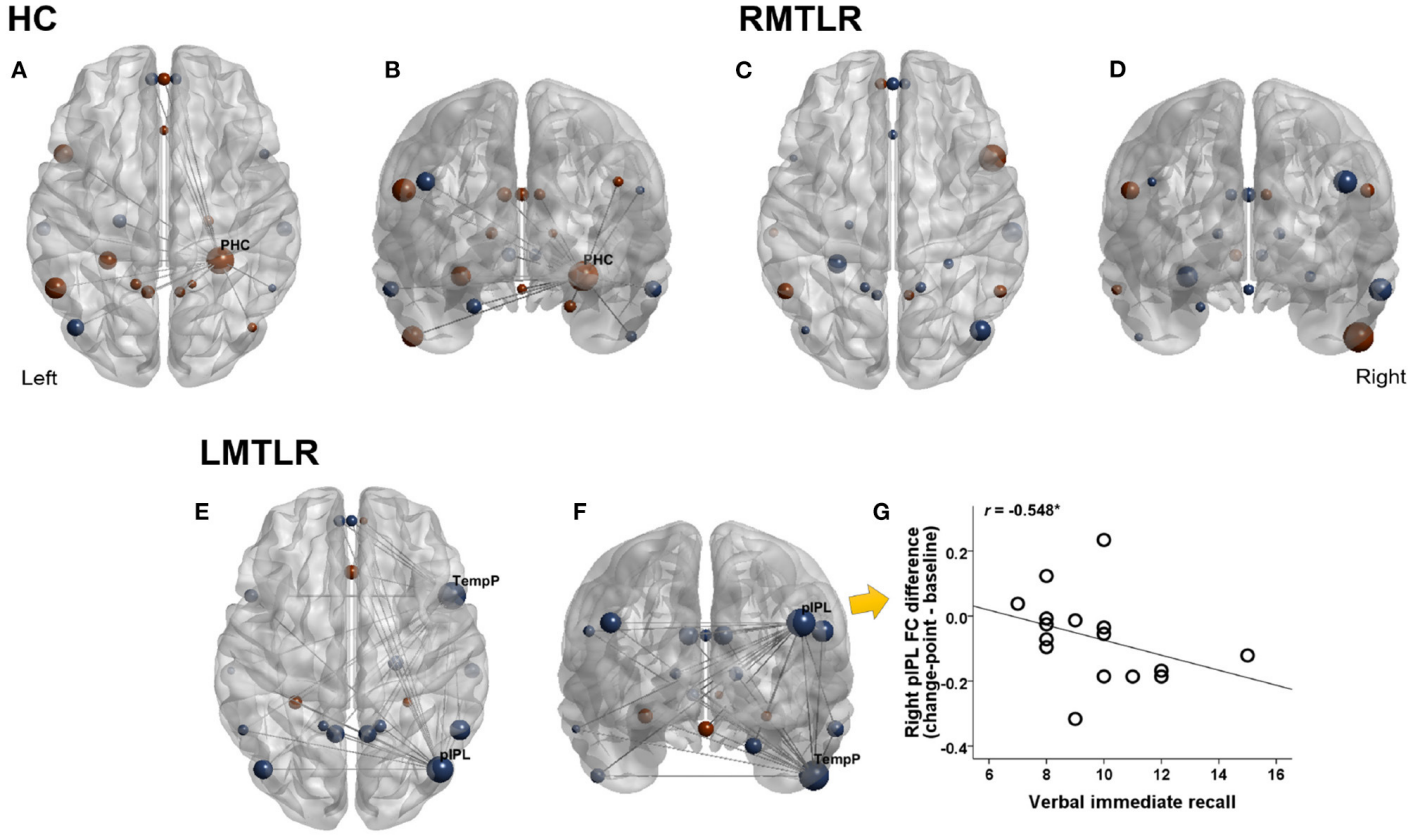
Changes of functional connectivity during pre- and post-encoding (after change-point) resting. Red color indicates increased FC and blue indicates decreased FC during post-encoding (Post-encoding minus Pre-encoding baseline). Size of circle indicates relative magnitude of FC changes in all ROIs in HC (healthy controls), LMTLR (left medial temporal lobe), and RMTLR (right MTLR). We displayed functional connections with gray line only in ROIs that showed significant (*p* < 0.05) or trend (*p* < 0.1) of changes. **(A,C,E)** Top view. **(B,D,F)** Front view. **(G)** Relationships of FC differences to memory capacity. ^*^Significant at 0.05 level.

Changes of FC after change-point and pre-encoding baseline are compared within each subject group by using the Wilcoxon signed rank test (Figure 2). FC without considering change-point estimation (whole-segment of post-encoding period) are also compared with baseline FC. FC values in areas that survived are presented in Table 7. In the HC group, the FC of the right parahippocampal cortex (PHC) shows a tendency to increase after the change-point (*p* = 0.067) and significantly increased in whole-segment of post-encoding rest (*p* < 0.05). In RMTLR group, none of ROIs shows significant FC differences. In the LMTLR group, although not statistically significant, the FC of the right temporal pole (TempP) shows a tendency to decrease after change-point (*p* = 0.088). In this ROI, whole-segment comparisons shows significant difference (*p* < 0.05). Interestingly, the FC of the right posterior inferior parietal lobule (pIPL) is significantly decreased after the change-point (*p* < 0.05) but shows no differences with the whole-segment of post-encoding rest (*p* = 0.642). Moreover, the FC difference (after change-point – baseline) in the right pIPL is negatively correlated with verbal immediate memory scores of the standard neuropsychological Rey-Kim memory test in LMTLR group (*r* = −0.548, *p* = 0.028; Spearman's correlation; Figure 2. There are no other significant clinical correlations of FC, to memory scores in either after-change point or whole-segment comparisons.

**Table 7:** Functional connectivity during pre– and post–encoding rest.

	**ROI**	**Pre-encoding**	**Post-encoding**	**Post-encoding**
		**baseline**	**(after change-point)**	**(whole-segment)**
HC	Right parahippocampal cortex	0.209 (0.1308)	0.280 (0.1482)^†^	0.280 (0.1290)
LMTLR	Right posterior inferior parietal lobule	0.238 (0.1065)	0.171 (0.1451)^*^	0.238 (0.0897)
	Right temporal pole	0.211 (0.0800)	0.140 (0.1567)^†^	0.170 (0.0873)^*^

Data presented as mean (SD). Pre– vs. Post–encoding differences(^†^ p < 0.01, ^*^p < 0.05).

**Figure 3:**
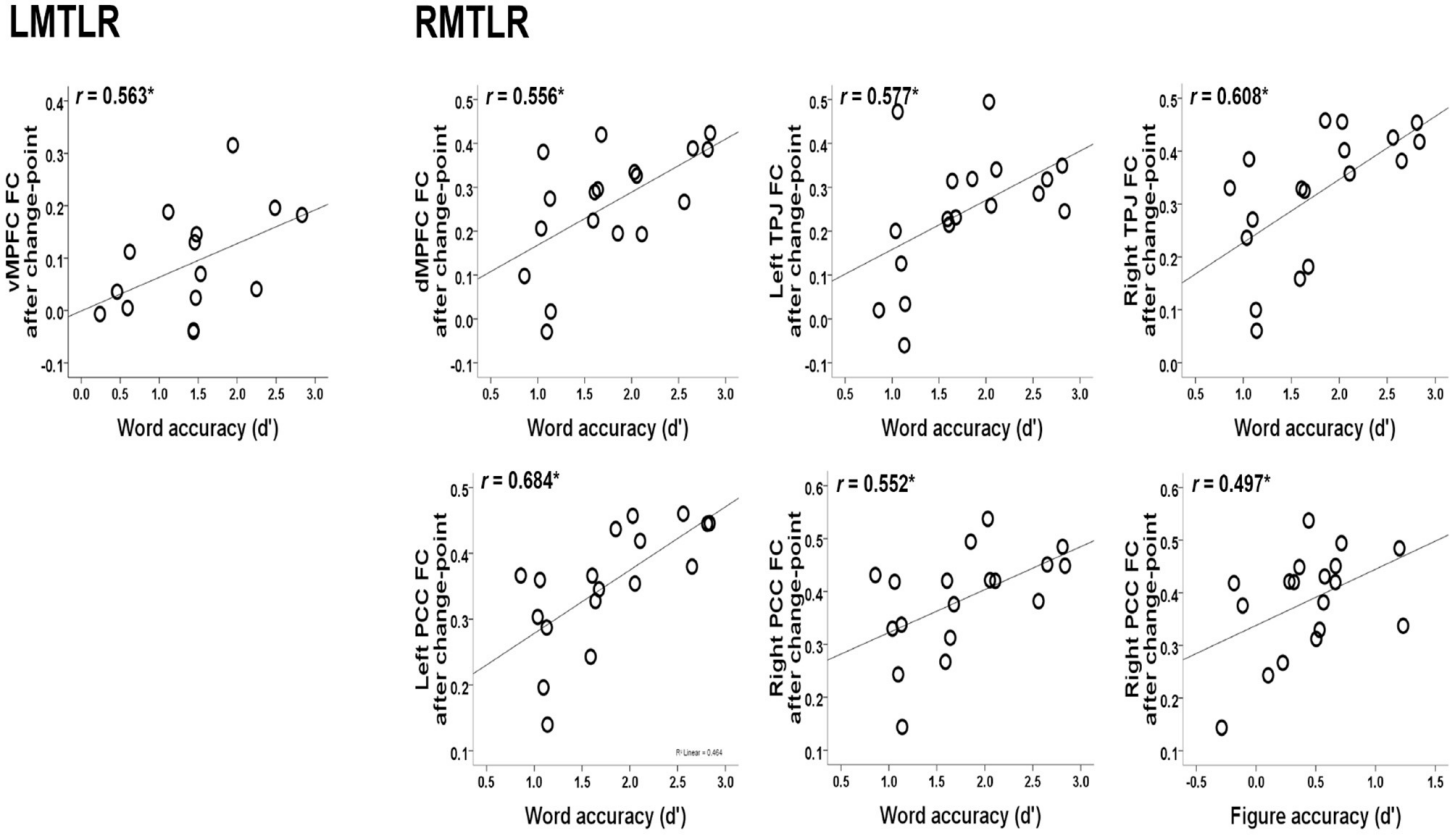
Relationships between the behavioral accuracy of in-scanner memory task (words, figures) and the FC values of post-encoding resting after change-point. *Significant at 0.05 level.

The relationships between the behavioral accuracy (d-prime) of the in-scanner memory task and the FC values of post-encoding rest after change-point are also analyzed. Our previous study describes behavioral accuracy in detail (Jeong et al., 2019). Figure 3 shows the significant correlations. The FC of the ventral medial prefrontal cortex (vMPFC) is positively correlated with word accuracy in LMTLR (*r* = 0.563, *p* < 0.05). In RMTLR, word accuracy is positively correlated with the FC of the dorsal medial prefrontal cortex (dMPFC; *r* = 0.556), left and right posterior cingulate cortex (PCC; *r* = 0.684, *r* = 0.552), and left and right temporal parietal junction (TPJ; *r* = 0.577, *r* = 0.608) (*p* < 0.05 for all). Figure accuracy is positively correlated with the FC of the right PCC in the RMTLR group (*r* = 0.497, *p* < 0.05).

## 5. Discussion

In this paper we develop a new procedure that can be used to determine a change-point in a individual FC system, using a function of maximum eigenvalues of suitably scaled covariance matrices. The proposed method is applicable to either resting-state or task fMRI studies providing individualized change-point estimation. The method yields individualized change-point estimation results, but it permits to group-level estimation. Our change-point estimation method generally works well in simulations and shows improved performance over the previous approach. Our work avoids the use of a sliding window usually applied to correlation measures, and reinforces the notion that time-varying techniques are required to study FC. Our results stress the importance of taking the change-point into account to characterize dynamic FC in order to describe how “information” is integrated and changed over time.

In this study, we applied change-point estimation analysis to post-encoding rest fMRI data of MTLR patients in order to evaluate the applicability and usefulness of the proposed method. We found that both segment after change-point and whole-segment of resting-state data after memory encoding detect similar FC changes. In HC, the MTL area (PHC) shows increased FC after memory encoding which is consistent with previous findings of memory consolidation studies (Tambini et al., 2010; Staresina et al., 2013). In LMTLR patients, the FC of the right pIPL and TempP decreased after memory encoding. Importantly, right pIPL is only detected when we use post-encoding data after the change-point, but not detected with the whole-segment of data. Moreover, the magnitude of FC changes in the right pIPL is associated with verbal memory capacity of LMTLR patients. Patients who displayed more reduction in FC values have better verbal memory. Recent studies suggest that the posterior parietal cortex contributes to memory consolidation (Brodt et al., 2016, 2018). Therefore, we speculate that DMN areas other than damaged MTL, especially the right pIPL, may play a compensatory role for the verbal memory function of resected left MTL. In summary, by applying change-point analysis, we can reveal additional information on the memory consolidation network in an MTL resected brain. Further studies with other data sets are warranted to ensure the general use of this change-point estimation analysis of fMRI data.

The other notable contribution of our work is that we develop a generalized multivariate extension to use an eigenvalue system based on RMT. While previous approaches have largely targeted univariate approaches examining a single time series or a single measure of association, our method use full information of connectivity via the eigenvalue system. Another interesting aspect of our change-point detection is the quantitative comparison before and after change-point. It reveals some properties of FC after change occurs. Different analytic approaches provides different perspective information on fMRI data, which necessitates integrating knowledge gained through the variety of models for the understanding of dynamic FC.

One limitation of the method is that the simulation results depend on the underlying covariance structure. When there are more change-points, simultaneous multiple change-points estimation procedure should be used. Further study with this extension is expected when there are multiple change-points in FC.

## Data Availability Statement

The datasets generated for this article are not readily available because: it can be available only if requested to the third author. Requests to access the datasets should be directed to Chun Kee Chung, chungc@snu.ac.kr.

## Ethics Statement

The studies involving human participants were reviewed and approved by Institutional Review Board of Seoul National University Hospital. The patients/participants provided their written informed consent to participate in this study.

## Author Contributions

Statistical method was developed and simulation work was done by JK. Epilepsy data were collected by CC. Epilepsy data analyses and interpretations were conducted by WJ. All authors contributed to the article and approved the submitted version.

## Conflict of Interest

The authors declare that the research was conducted in the absence of any commercial or financial relationships that could be construed as a potential conflict of interest.
